# Non-pain-related CRF1 activation in the amygdala facilitates synaptic transmission and pain responses

**DOI:** 10.1186/1744-8069-9-2

**Published:** 2013-02-15

**Authors:** Guangchen Ji, Yu Fu, Hita Adwanikar, Volker Neugebauer

**Affiliations:** 1 Department of Neuroscience & Cell Biology, The University of Texas Medical Branch, 301 University Blvd, Galveston, Texas, 77555-1069, USA

**Keywords:** Amygdala, Pain, CRF, Synaptic transmission, Vocalizations

## Abstract

**Background:**

Corticotropin-releasing factor (CRF) plays an important role in affective states and disorders. CRF is not only a “stress hormone” but also a neuromodulator outside the hypothalamic-pituitary-adrenocortical (HPA) axis. The amygdala, a brain center for emotions, is a major site of extrahypothalamic expression of CRF and its G-protein-coupled receptors. Our previous studies showed that endogenous activation of CRF1 receptors in an arthritis pain model contributes to amygdala hyperactivity and pain-related behaviors. Here we examined the synaptic and behavioral effects of CRF in the amygdala of normal animals in the absence of tissue injury or disease.

**Results:**

Whole-cell patch-clamp recordings of neurons in the latero-capsular division of the central nucleus of the amygdala (CeLC) in brain slices from normal rats showed that CRF (0.1-10 nM) increased excitatory postsynaptic currents (EPSCs) at the “nociceptive” parabrachio-amygdaloid (PB-CeLC) synapse and also increased neuronal output. Synaptic facilitation involved a postsynaptic action and was blocked by an antagonist for CRF1 (NBI27914, 1 μM) but not CRF2 (astressin-2B, 1 μM) and by an inhibitor of PKA (KT5720, 1 μM) but not PKC (GF109203X, 1 μM). CRF increased a latent NMDA receptor-mediated EPSC, and this effect also required CRF1 and PKA but not CRF2 and PKC. Stereotaxic administration of CRF (10 μM, concentration in microdialysis probe) into the CeLC by microdialysis in awake rats increased audible and ultrasonic vocalizations and decreased hindlimb withdrawal thresholds. Behavioral effects of CRF were blocked by a NBI27914 (100 μM) and KT5720 (100 μM) but not GF109203x (100 μM). CRF effects persisted when HPA axis function was suppressed by pretreatment with dexamethasone (50 μg/kg, subcutaneously).

**Conclusions:**

Non-pain-related activation of CRF1 receptors in the amygdala can trigger pain-responses in normal animals through a mechanism that involves PKA-dependent synaptic facilitation in CeLC neurons independent of HPA axis function. The results suggest that conditions of increased amygdala CRF levels can contribute to pain in the absence of tissue pathology or disease state.

## Background

Corticotropin releasing factor (CRF) plays an important role in emotional processes and neuropsychiatric disorders such as anxiety, depression and addiction [1-5]. CRF acts on CRF1 and CRF2 receptors that can couple to multiple G proteins to activate a number of intracellular effectors such as protein kinase A and C (PKA and PKC) [6-9]. Antagonists selective for CRF1 have been developed for clinical trials. Whereas an open-label trial study reported effectiveness in human patients with major depressive disorder [10], evidence from several clinical trials now suggests that CRF1 antagonists may be more useful in certain anxiety disorders such as posttraumatic stress disorder (PTSD) and in addiction disorders [5].

The amygdala, a major site of extra-hypothalamic expression of CRF and its receptors, has emerged as a key element of the circuitry through which CRF acts as a neuromodulator outside the hypothalamic-pituitary-adrenocortical (HPA) axis to mediate behavioral responses to stressors [1,2,4,8,9,11-15]. CRF-containing neurons are found in the central nucleus of the amygdala (CeA), which serves as the output nucleus for major amygdala functions [16-18]. CRF neurons in the CeA project to widespread regions of the basal forebrain and brain stem [12] and in turn receive input from calcitonin gene-related peptide (CGRP) terminals [19] arising from brainstem neurons in the lateral parabrachial area [20]. The latero-capsular division of the central nucleus (CeLC) is delineated by these CGRP containing fibers from the parabrachial area (PB) that provide unfiltered nociceptive information to the CeLC as part of the spino-parabrachio-amygdaloid pain pathway [21,22]. Pain-related plasticity at the PB-CeLC synapse has been shown in different pain models [23-30]. CeLC plasticity drives pain-related emotional-affective responses and anxiety-like behavior [21,22].

Accumulating evidence from biochemical [31-33], behavioral [29,34-36] and electrophysiological studies [29,37,38] suggests that CRF in the amygdala, particularly in the CeA, plays an important role in pain modulation and pain-related affect. These studies showed increased CRF mRNA expression in the CeA in models of visceral [31] and neuropathic [32,33] pain. Inhibition of CRF-binding protein in the CeA to increase availability of CRF increased mechanical sensitivity in neuropathic animals and this effect was blocked by a non-selective CRF receptor antagonist [36]. Microinjections of a non-selective CRF receptor antagonist into the CeA reduced hyperalgesia (tail flick test) associated with morphine withdrawal without affecting plasma corticosterone responses [34]. Blockade of CRF1 receptors in the CeA inhibited pain- and anxiety-like behaviors in a model of arthritic pain [29,35] whereas a non-selective CRF receptor antagonist had no effect in a neuropathic pain model [36]. A CRF1 receptor antagonist inhibited pain-related central sensitization and attenuated synaptic plasticity in CeA neurons [29,37].

Little is known about the effects of CRF administration to the amygdala (CeA) on pain-related behavior and underlying neuronal mechanisms under normal conditions in the absence of disease or injury. Our previous studies suggest that the CRF system is not active under normal conditions because CRF receptor antagonists had no effect on their own [29,37]. Intra-CeA application of CRF increased responsiveness of CeLC neurons in normal animals but also had inhibitory effects at higher concentrations [38]. It is not clear if these effects were due to direct actions on CeLC neurons or in the amygdala network. In brain slices, CRF inhibited the slow afterhyperpolarization following evoked action potential firing, which would increase excitability [39].

Here we determined the synaptic effects of CRF on CeLC neurons, contribution of CRF1 and CRF2 receptors, signaling mechanisms and behavioral consequences under normal conditions. The significance of this study is that the amygdala CRF system is not only engaged in conditions of pain and anxiety disorders but can by itself trigger pain in the absence of any injury or disease.

## Results

Neurons in the latero-capsular division of the CeA (CeLC) with non-accommodating spike firing properties were recorded in brain slices from untreated normal rats. These are Type A projection neurons that target brainstem and forebrain areas [21,40,41] Neurons were selected that showed an excitatory synaptic response to electrical stimulation of presumed parabrachial (PB) afferents (PB-CeLC synapse; see Methods) as described in our previous studies [25,29]. These CeLC neurons form the “nociceptive amygdala” [21,22]. They develop CRF1 receptor-dependent central sensitization and synaptic plasticity in an arthritis pain model [29,37], but synaptic and behavioral effects of non-pain-related CRF increases in the CeLC under normal conditions remain to be determined.

Since characteristics of CeLC neurons with PB input have been described in detail in our previous studies [22] for recent references see [30,42,43] this report will focus on the novel findings related to the exogenous application of CRF to the amygdala. The first part of this study examined CRF effects on synaptic transmission and the involvement of CRF1 versus CRF2 receptors and PKA versus PKC in brain slices from normal animals. Only one or two brain slices per animal were used; one neuron was recorded in each slice, and a fresh slice was used for each new experimental protocol. Numbers in the manuscript refer to the number of neurons tested for each parameter. The second part of this study takes these findings to behavioral level, measuring pain-like responses (vocalizations and reflexes) in awake animals.

### Facilitation of synaptic transmission by CRF through CRF1 receptors

**Figure 1:**
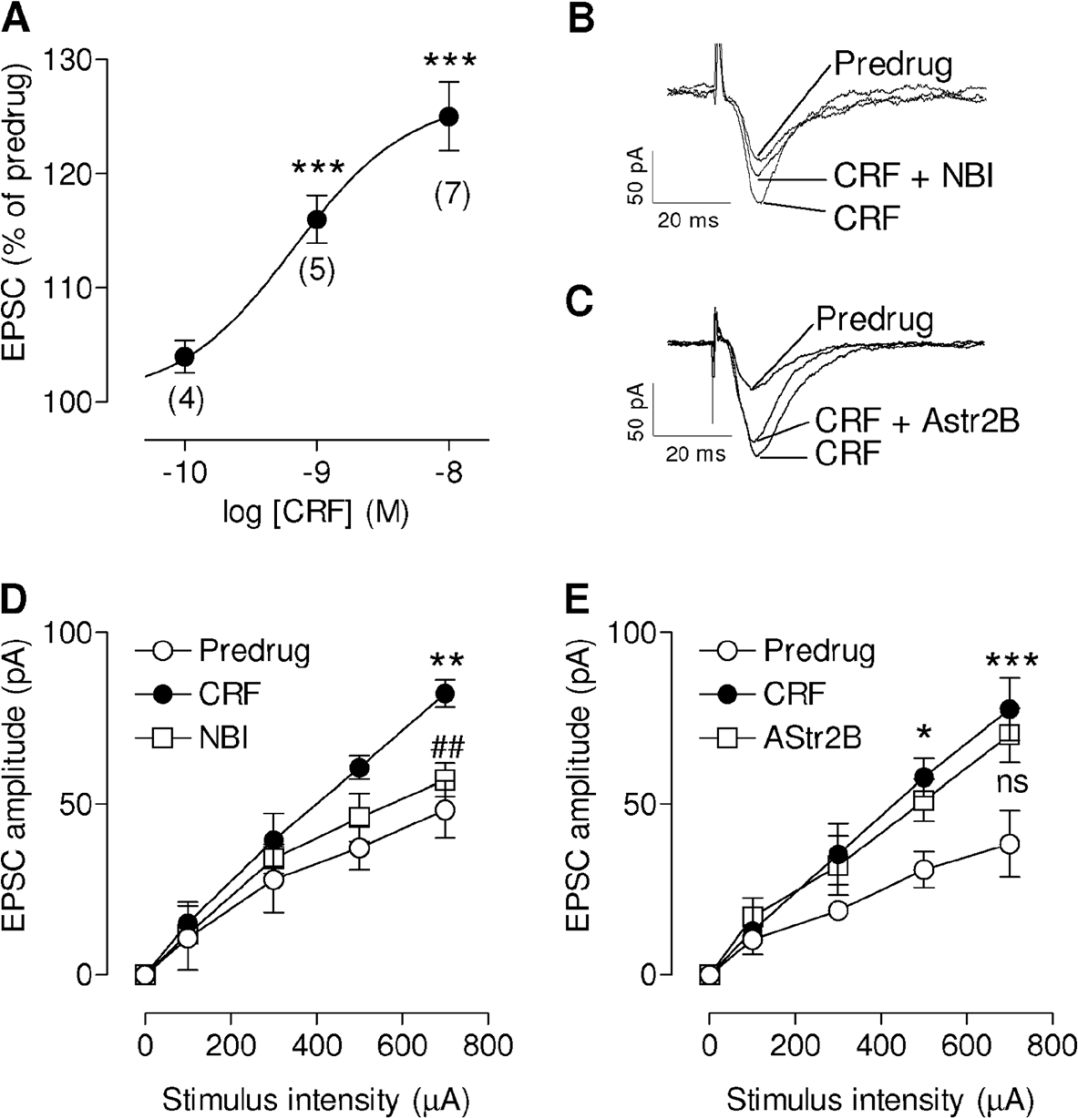
**CRF enhances synaptic transmission in the CeLC in slices from normal animals.** (**A**) Concentration-response relationship of CRF effects on monosynaptic EPSCs (numbers of neurons tested with each concentration are indicated). Peak amplitudes were averaged for each concentration of CRF and expressed as percent of predrug control (set to 100%). Concentration-response curve was obtained by non-linear regression analysis using the formula *y* = *A* + (*B* − *A*)/[1 + (10^*C*^/10^*X*^)^*D*^], where *A* is the bottom plateau, *B* top plateau, *C* = log(EC_50_), and *D* is the slope coefficient (GraphPad Prism software). *** P < 0.001, Bonferroni posttests compared to predrug. (**B-E**) Synaptic facilitation by CRF (10 nM, 12 min) was blocked by co-administration of an antagonist for CRF1 (NBI27914, NBI; 1 μM, 12 min) but not for CRF2 (astressin-2B, AStr2B; 1 μM, 12 min). (**B, C**) Monosynaptic EPSCs recorded in ACSF (Predrug), during CRF, and during CRF together with NBI27914 (**B**) or astressin-2B (**C**). Individual traces are the average of 8–10 EPSCs. (**D**) CRF increased input–output function significantly (n = 7 neurons). NBI27914 (n = 5) decreased the effect of CRF. Input–output curves were generated by plotting peak EPSC amplitude (pA) as a function of afferent fiber volley stimulus intensity (μA). (**E**) Astressin-2B (n = 5) had no significant (ns) effect on CRF-induced synaptic facilitation (n = 7). *,**,*** P < 0.05, 0.01, 0.001, Bonferroni posttests compared to predrug. ^##^ P < 0.01, Bonferroni posttests compared to CRF. CeLC neurons were recorded at −60 mV in slices from naïve untreated animals. Symbols and error bars represent means ± SEM.

Superfusion of the brain slice with CRF (0.1-10 nM) increased excitatory transmission at the PB-CeLC synapse concentration-dependently in 7 of 10 neurons (P < 0.001, one-way ANOVA; Figure 1A). Monosynaptic excitatory postsynaptic currents (EPSCs) were evoked by electrical stimulation of presumed PB afferents [25,28,29]. Baseline EPSCs at the PB-CeLC synapse recorded at resting membrane potential are mediated by non-NMDA receptors since they persist in the presence of NMDA receptor blockade as shown in our previous studies [28] and confirmed by others [24]. CRF enhanced the input–output function of the PB-CeLC synapse significantly (Figure 1D,E, n = 7 neurons in each sample; P < 0.0001, F_1,60_ = 12.94 and 19.02, respectively; two-way ANOVA). Input–output relationships were obtained by increasing stimulus intensities to measure EPSC peak amplitude as a function of afferent fiber volley stimulus intensity for each neuron. Synaptic facilitation by CRF (10 nM) was blocked by co-administration of a CRF1 receptor antagonist (NBI27914, 1 μM, n = 5 neurons; P < 0.01, Bonferroni posttest; Figure 1B,D) but not by a CRF2 receptor antagonist (astressin-2B, 1 μM, n = 5 neurons; P > 0.05, Bonferroni posttest; Figure 1C,E). These data show the presence of functional CRF1 receptors in the CeLC under normal conditions and the ability of CRF to facilitate excitatory synaptic transmission.

### CRF acts postsynaptically to increase synaptic transmission

**Figure 2:**
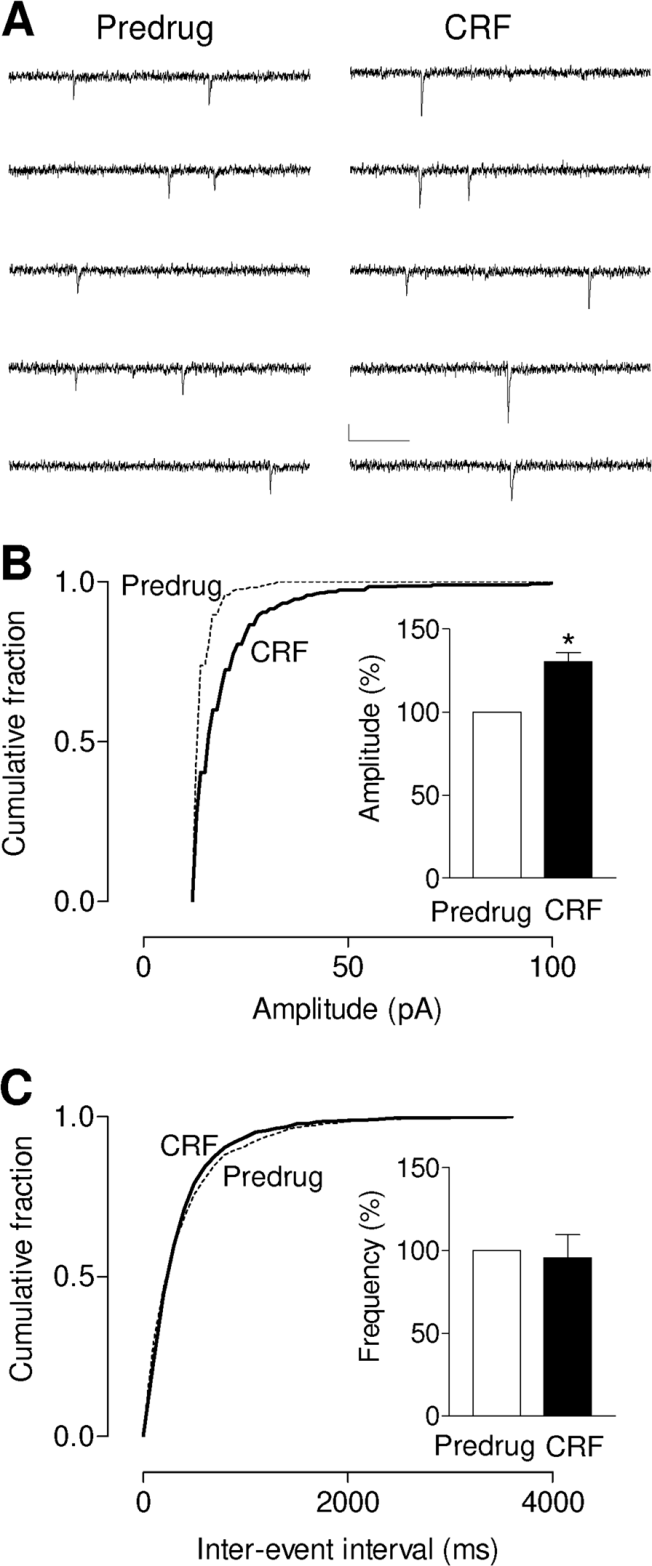
**Post- rather than pre-synaptic effect of CRF.** (**A**) Original current traces of miniature EPSCs (mEPSCs) recorded in the presence of TTX (1 μM) in one CeLC neuron before (Predrug) and during CRF (10 nM). Scale bars, 20 pA, 200 ms. CRF (10 nM, 12 min) increased amplitude (**B**) but not frequency (**C**) of mEPSC significantly (cumulative inter-event interval distribution for individual neuron, P < 0.0001, Kolmogorov–Smirnov test; mean frequency, n = 5 neurons, P < 0.05, paired t-test). Data for each neuron were obtained from 2 predrug recording periods and 2 recording periods during CRF (5 min each period). Bar histograms show means ± SEM expressed as percent of predrug control (set to 100%). Statistical analysis was done using raw data.

To determine the synaptic site of action of CRF in the CeLC we analyzed amplitude and frequency distribution of miniature EPSC (mEPSC) in the presence of TTX, which is a well-established electrophysiological method to distinguish pre- and postsynaptic mechanisms [44]. Presynaptic effects at the transmitter release site change mEPSC frequency whereas postsynaptic membrane effects alter mEPSC amplitude (quantal size). CRF (10 nM, 12 min) increased amplitude (Figure 2B) but not frequency (Figure 2C) of mEPSCs in the presence of TTX (1 μM), causing a significant shift of the cumulative mEPSC amplitude distribution towards larger amplitudes (P < 0.0001, Kolmogorov-Smirnov test; Figure 2B) and increasing the mean mEPSC amplitude in the sample of neurons significantly (n = 5, P < 0.05, paired t-test; Figure 2B, bar histogram). CRF had no significant effect on the frequency of mEPSCs (see cumulative inter-event interval distribution, P > 0.05, Kolmogorov-Smirnov test; mean frequency for the sample of neurons, n = 5, P > 0.05, paired t-test; Figure 2C). The results are consistent with a postsynaptic site of action of CRF.

### CRF increases CeLC output (depolarization-induced spiking)

**Figure 3:**
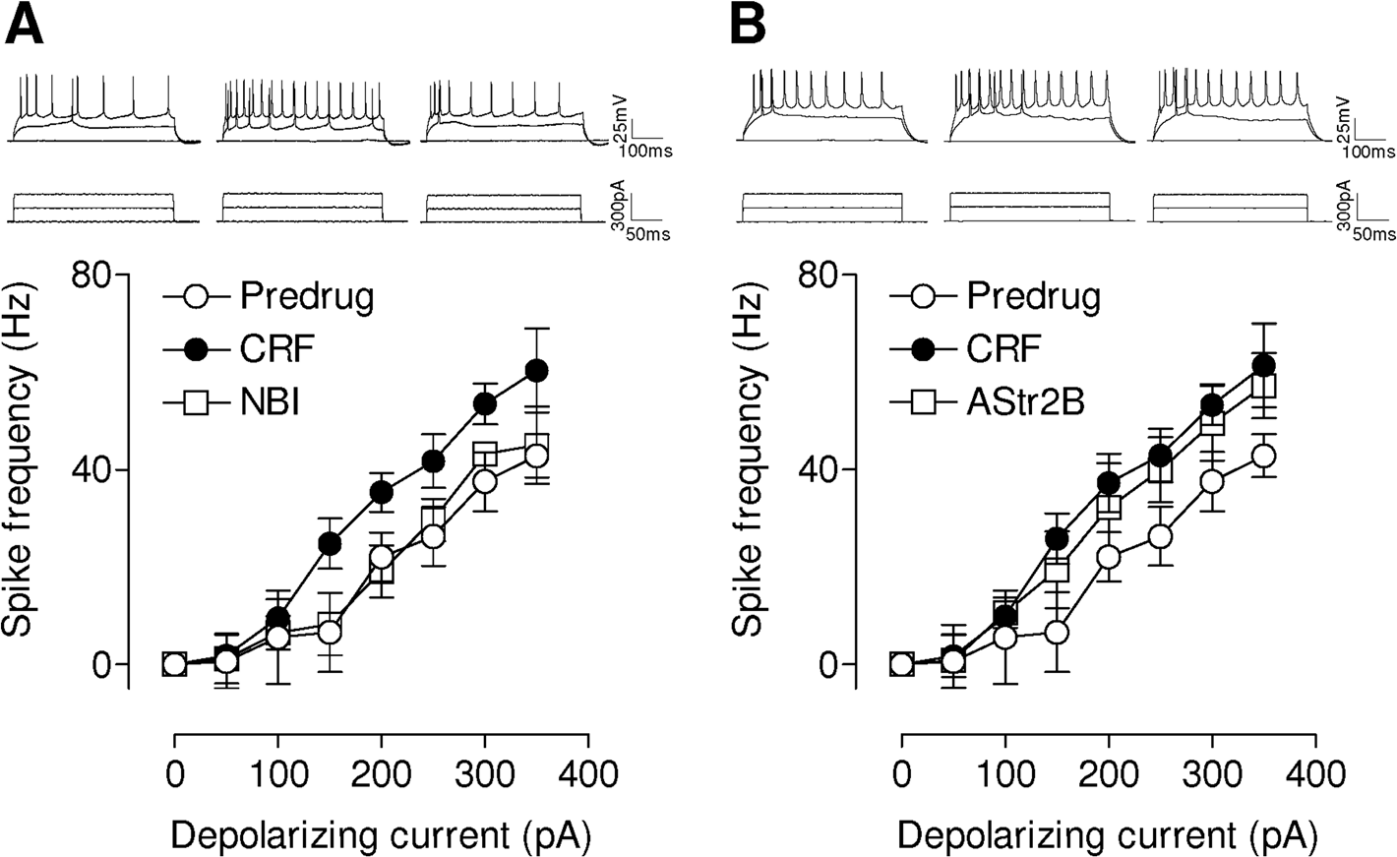
**CRF increases depolarization-induced spiking.** Whole-cell current-clamp recordings of action potentials generated by intracellular (through the patch electrode) injections of depolarizing current pulses (500 ms) of increasing magnitude (in 50 pA steps) from a membrane potential of −60 mV. (**A, B**) Upper traces show action potential firing rate increased during superfusion of CRF (10 nM, 12 min). Lower traces show depolarizing current steps. Graphs show input–output functions (frequency-current [F-I] relationships) averaged for each sample of neurons. CRF increased F-I relationships significantly (**A,** n = 5 neurons; P < 0.0001, F_1,56_ = 14.87; **B,** n = 5 neurons; P < 0.0001, F_1,56_ = 15.68; compared to predrug, two-way ANOVA). (**A**) A CRF1 receptor antagonist (NBI27914, NBI, 1 μM, n = 5) blocked the effect of CRF significantly (P < 0.0001, F_1,56_ = 14.53, two-way ANOVA). (**B**) A CRF2 receptor antagonist (astressin-2B, AStr2B, 1 μM, n = 5) had no significant effect (P > 0.05, F_1,56_ = 0.89, compared to CRF, two-way ANOVA). Symbols and error bars represent means ± SEM.

Action potentials were evoked under current-clamp mode by direct intracellular current injections of increasing magnitude through the patch electrode (Figure 3). Input–output functions of neuronal excitability (frequency-current [F-I] relationships) were obtained by averaging the frequency of action potentials evoked at each current intensity. CeLC neurons were regular-spiking and showed no accommodation of action potential firing in response to sustained depolarization, which are characteristics of Type A projection neurons [40,41]. CRF (10 nM, 12 min) significantly increased the input–output function of CeLC neurons (Figure 3A,B, n = 5 neurons in each sample; P < 0.0001, F_1,56_ = 14.87 and 15.68, respectively, two-way ANOVA). A CRF1 receptor antagonist (NBI27914, 1 μM, 12 min, n = 5) blocked the effect of CRF (P < 0.0001, F_1,56_ = 14.53, two-way ANOVA; Figure 3A). In contrast, a CRF2 receptor antagonist (astressin-2B, 1 μM, n = 5) had no significant effect (P > 0.05, F_1,56_ = 0.89, two-way ANOVA) hence the excitatory action of CRF continued. The persistence of CRF effects in the presence of the CRF2 antagonist argues against the loss of effectiveness of prolonged CRF application (30 min), e.g., through desensitization, as an explanation for the inhibitory effect of the CRF1 antagonist. Thus, CRF increases CeLC output through CRF1 receptor activation.

### Inhibition of PKA, but not PKC, blocks CRF-induced synaptic facilitation

**Figure 4:**
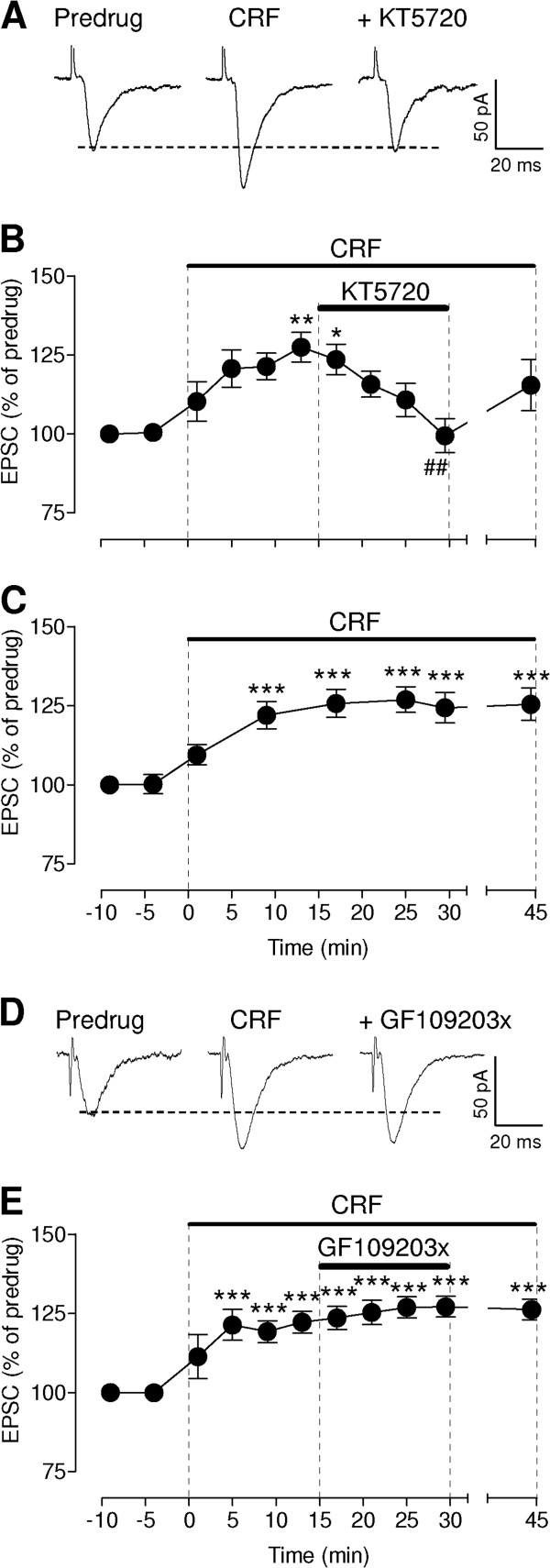
**Inhibition of PKA, but not PKC, blocks CRF-induced synaptic facilitation.** (**A**) Original recordings of monosynaptic EPSCs (average of 8–10 traces). Facilitatory effects of CRF (10 nM) were blocked by co-administration of a PKA inhibitor (KT5720, 1 μM). (**B**) Summary of time course data for the sample of CeLC neurons (n = 5). Peak amplitudes of EPSCs recorded during drug application were expressed as percent of predrug control values (set to 100%). Symbols and error bars represent means ± SEM. *,** P < 0.05, 0.01, compared to predrug before CRF; ^##^ P < 0.01, compared to the data point immediately before KT5720 application; ANOVA with Bonferroni posttests. (**C**) Time course data for prolonged application of CRF without a PKA inhibitor (n = 6). Display as in (**B**). *** P < 0.001, compared to predrug, Dunnett’s multiple comparison tests. (**D**) Individual traces (average of 8–10) of monosynaptic EPSCs show that the facilitatory effect of CRF (10 nM) was not blocked by co-administration of a PKC inhibitor (GF109203x, 1 μM). (**E**) Time course data for GF109203x effects (n = 5). Significant facilitation by CRF persisted during coapplication of GF109203x. Display as in (**B**). *** P < 0.001, compared to predrug, Dunnett’s multiple comparison tests. Statistical analysis was performed on raw data.

CRF receptors can couple to a number of signaling pathways including cAMP-PKA activation (see Background). PKA, but not PKC, plays a critical role in pain-related plasticity in the CeLC [45]. Therefore, we tested the hypothesis that CRF-induced synaptic facilitation depends on PKA. Co-application of a selective PKA inhibitor (KT5720, 1 μM) decreased synaptic facilitation by CRF significantly (Figure 4A, B, n = 5, P < 0.01, compared to the data point immediately before KT5720 application, Bonferroni posttests). The effect of KT5720 was reversible. Prolonged application of CRF alone (10 nM) resulted in continued synaptic facilitation (Figure 4C, n = 6, P < 0.001, compared to predrug, Bonferroni posttests). In contrast, a selective PKC inhibitor (GF109203x, 1 μM) had no significant effect on CRF-induced synaptic facilitation (Figure 4D, E, n = 5, P > 0.05, Dunnett's multiple comparison test). The data show an important contribution of PKA to CRF1 receptor-mediated synaptic facilitation.

### CRF increases NMDA receptor-mediated transmission through CRF1 and PKA

**Figure 5:**
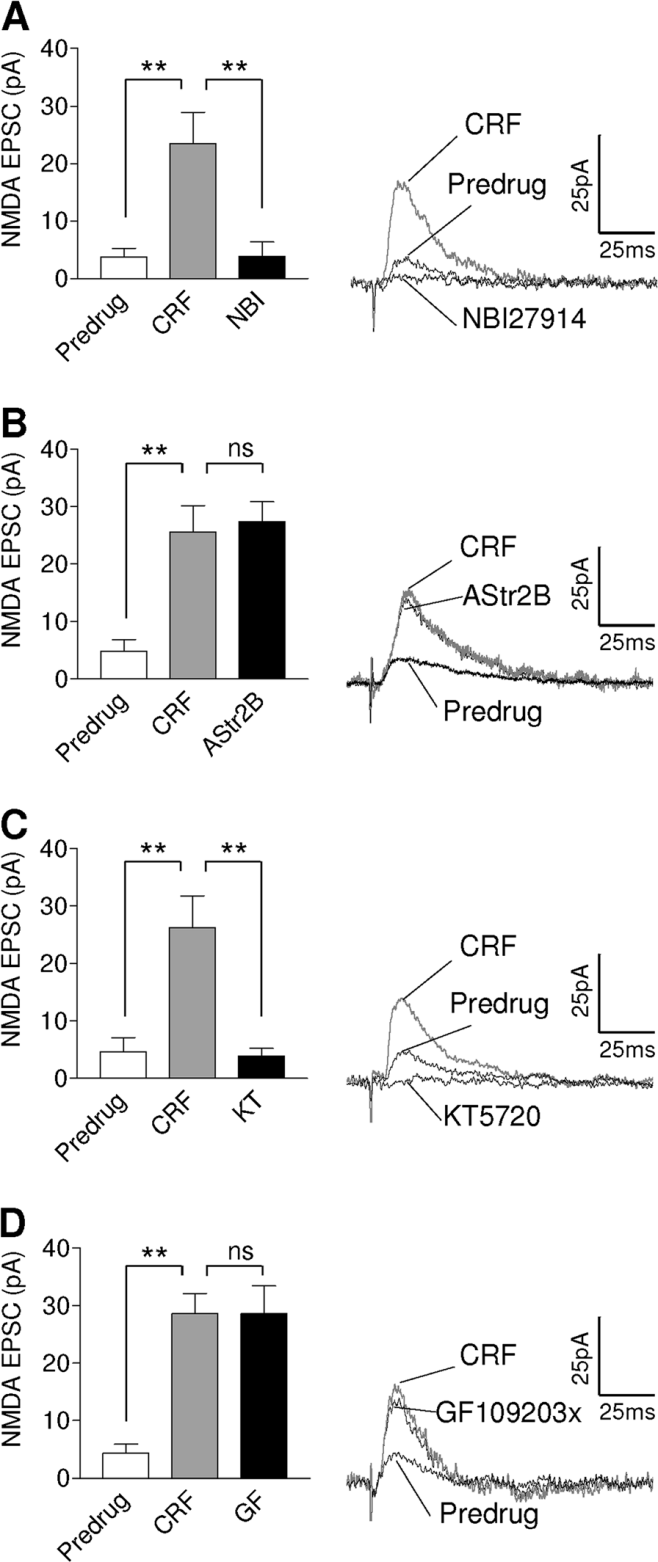
**CRF increases NMDA receptor-mediated transmission through CRF1 and PKA.** (**A-D**) CRF (10 nM) increased a small pharmacologically (bicuculline, 30 μM; NBQX, 20 μM) isolated NMDA component recorded at a holding potential of +20 mV that was blocked by an NMDA receptor antagonist (AP5, 50 μM; not shown). Bar histograms show averaged data (mean ± SE). Individual traces are the average of 8–10 EPSCs recorded at +20 mV. (**A**) A CRF1 receptor antagonist (NBI27914, 1 μM, n = 5) inhibited the facilitatory effect of CRF. (**B**) A CRF2 receptor antagonist (astressin-2B, AStr2B, 1 μM, n = 5) had no effect. (**C**) A PKA inhibitor (KT5720, 1 μM, n = 5) blocked the CRF-induced facilitation. (**D**) A PKC inhibitor (GF109203x, 1 μM, n = 5) had no effect. Drugs were applied for 12–15 min. ** P < 0.01; ns (not significant) P > 0.05; ANOVA with Bonferroni posttests. Statistical analysis was performed on raw data.

PKA activation can increase NMDA receptor function in CeLC neurons, which plays an important role in synaptic plasticity associated with arthritis pain [28] but not neuropathic pain [24]. In addition, the facilitatory effect of another neuropeptide, calcitonin gene-related peptide (CGRP), on CeLC neurons depends on PKA-mediated activation of NMDA receptors [27,46]. Therefore, we examined the effect of CRF on NMDA receptor-mediated transmission at the presumed PB-CeLC synapse (Figure 5). CRF (10 nM) increased a small latent NMDA receptor-mediated synaptic response that was unmasked at a depolarized holding potential (+20 mV) in the presence of NBQX (20 μM) and bicuculline (30 μM) and was completely blocked by an NMDA receptor antagonist (AP5, 50 μM; not shown). An antagonist for CRF1 (NBI27914, 1 μM, n = 5, Figure 5A) but not CRF2 (astressin-2B, 1 μM, n = 5, Figure 5B) receptors inhibited the facilitation by CRF significantly (P < 0.01, Bonferroni posttest). An inhibitor of PKA (KT5720, 1 μM, n = 5, Figure 5C), but not PKC (GF109203x, 1 μM, n = 5, Figure 5D), blocked the CRF-induced facilitation of the NMDA EPSC significantly (P < 0.01, Bonferroni posttest). The data suggest that CRF can engage NMDA receptor-mediated synaptic transmission through a mechanism that involves CRF1 and PKA activation. It should be noted that our previous studies showed that CRF receptor antagonists and PKA and PKC inhibitors have no effect on their own under normal conditions [29,45]. These control experiments were not repeated here.

### CRF increases vocalizations and spinal reflexes through CRF1 receptors

Changes in PB-CeLC transmission and activity of CeLC neurons are positively correlated with pain-like behaviors [21,22]. Therefore we examined the behavioral consequences of CRF-induced facilitation of synaptic transmission. The effects of CRF administered into the CeLC on spinally (hindlimb withdrawal reflexes) and supraspinally (vocalizations) organized behaviors were measured in normal naïve animals. Thresholds for hindlimb withdrawal reflexes were determined by compressing the knee joint with gradually increasing stimulus intensities using a calibrated forceps whose output was displayed on an LCD screen (see Methods). Vocalizations in the audible (20 Hz to 16 kHz) and ultrasonic (25 ± 4 kHz) ranges reflect nocifensive and affective responses, respectively, to aversive stimuli [47]. Vocalizations evoked by brief (15 s) mechanical stimulation of the knee were recorded for a period of 1 min starting with the onset of the stimulus, using a computerized analysis system (see Methods) as described previously [47,48]. No apparent differences were found in this study for drug effects on vocalizations *during* stimulation and vocalization *after*-discharges [48]. Therefore, the total duration (sum of individual vocalization events) for the recording period is shown. Rats did not vocalize spontaneously in a control period of 5–10 min before stimulation.

**Figure 6:**
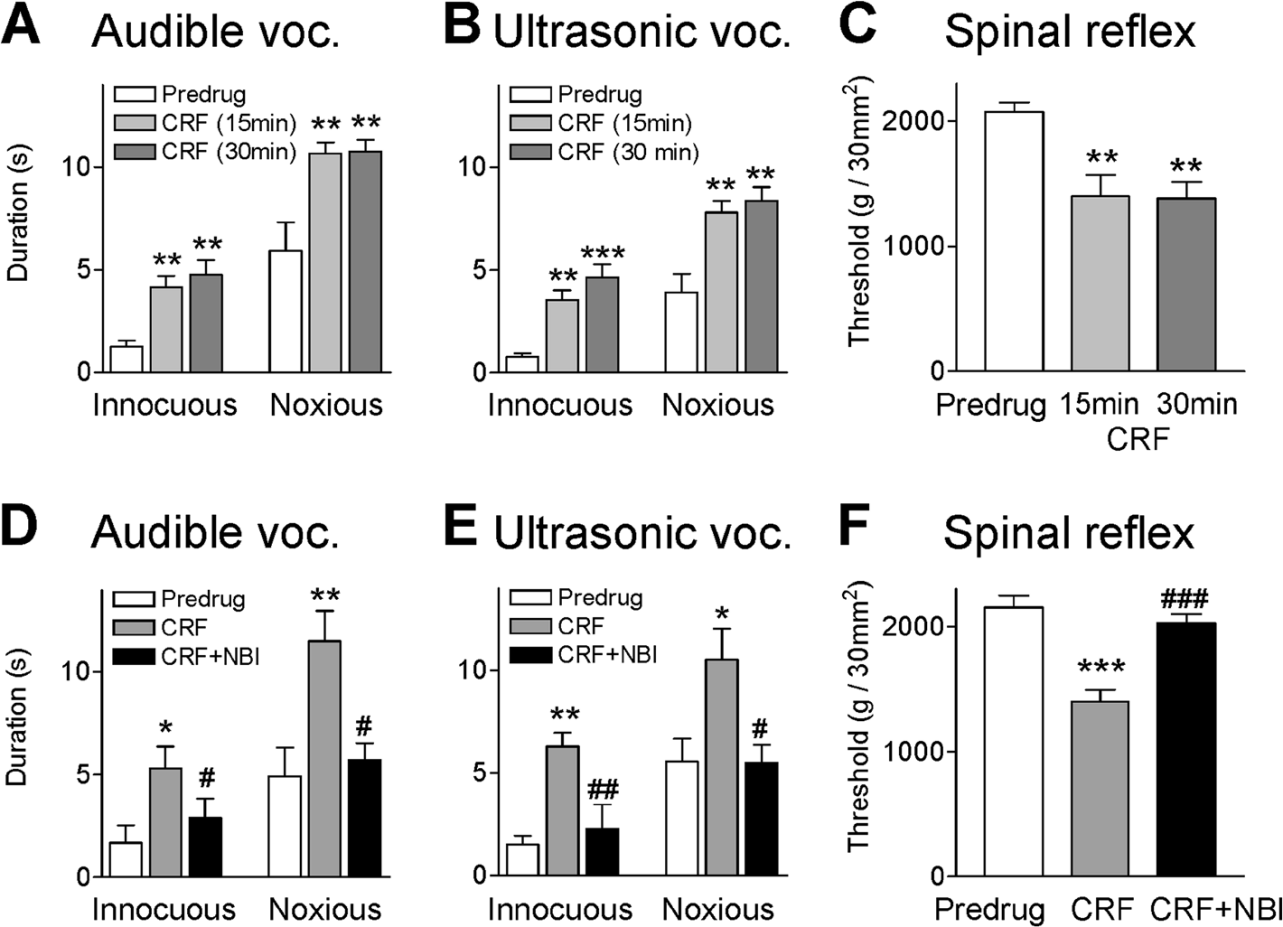
**A CRF1 receptor antagonist reverses CRF-induced increases of audible vocalizations and spinal reflexes.** Administration of CRF (1 μM, concentration in microdialysis fiber) into the CeLC increased the duration of audible (**A**) and ultrasonic (**B**) vocalizations (voc.) evoked by innocuous (500 g/30 mm^2^) and noxious (2000 g/30 mm^2^) stimulation of the knee (n = 6 rats). (**C**) CRF decreased reflex thresholds for mechanical stimulation of the knee (n = 5 rats). Behaviors were measured at 15 and 30 min of CRF administration. The effects of CRF persisted during continued administration. (**D,E**) Coapplication of a CRF1 receptor antagonist (NBI27914, 100 μM, concentration in microdialysis probe, 15 min) with CRF decreased CRF-enhanced audible and ultrasonic vocalizations significantly (n = 6 rats). (**F**) NBI27914 also inhibited the CRF effect on spinal reflex thresholds (n = 5 rats). Bar histograms show means ± SEM. *,**,*** P < 0.05-0.001, compared to predrug (ACSF) before CRF; ^#^,^##^,^###^ P < 0.05-0.001, compared to CRF (15 min time point); Bonferroni posttests.

Administration of CRF (1 μM, concentration in the microdialysis probe; 30 min) into the CeLC increased the duration of audible (Figure 6A) and ultrasonic (Figure 6B) vocalizations significantly (n = 6 in each group, P < 0.01-0.001 compared to predrug controls, Bonferroni posttests). CRF also decreased the threshold for hindlimb withdrawal reflexes significantly (n = 5, P < 0.01; Figure 6C). Predrug baseline measurements were made during administration of ACSF through the microdialysis probe as vehicle control. There was no difference between behavioral responses measured at 15 min and 30 min during continued CRF administration. This allowed us in subsequent experiments to coapply blockers with CRF starting 15 min after the onset of CRF administration. Coapplication of a CRF1 receptor antagonist (NBI27914, 100 μM, 15 min) into the CeLC decreased audible and ultrasonic vocalizations (n = 6) and spinal reflexes (n = 5) significantly (P < 0.05-0.001, compared to CRF alone at 15 min, Bonferroni posttests; Figure 6D-F). We did not test a CRF2 receptor antagonist because the electrophysiological effects of CRF on CeLC neurons in brain slices did not involve CRF2 receptors.

### Behavioral effects of CRF depend on PKA but not PKC

**Figure 7:**
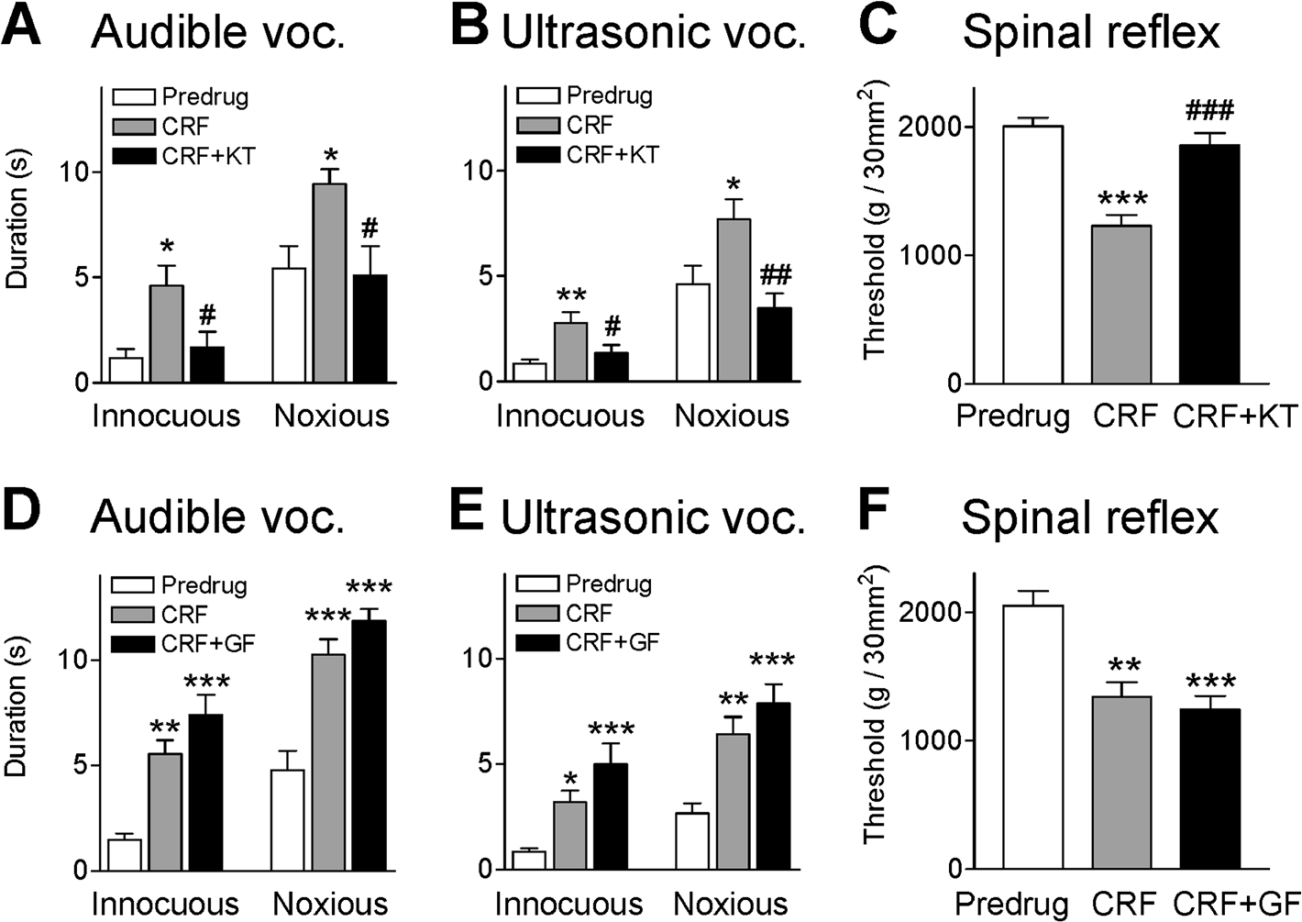
**Inhibition of PKA, but not PKC, blocks CRF effects on audible vocalizations and spinal reflexes.** (**A,B**) Coapplication of a PKA inhibitor (KT5720, 100 μM, concentration in microdialysis probe, 15 min) decreased the facilitatory effect of CRF on audible and ultrasonic vocalizations (voc.) significantly (n = 5 rats). (**C**) KT5720 also inhibited the CRF effect on spinal reflex thresholds (n = 5 rats). (**D,E,F**) Coapplication of a PKC inhibitor (GF109203x, 100 μM, concentration in microdialysis probe, 15 min) did not block the effects of CRF on audible and ultrasonic vocalizations (n = 6 rats) and on spinal reflex thresholds (n = 5 rats). Bar histograms show means ± SEM. *,**,*** P < 0.05-0.001, compared to predrug (ACSF) before CRF; ^#^,^##^,^###^ P < 0.05-0.001, compared to CRF (15 min time point); Bonferroni posttests.

Co-administration of a PKA inhibitor (KT5720, 100 μM, concentration in microdialysis probe) disrupted the facilitatory effects of CRF (1 μM) on audible (Figure 7A) and ultrasonic (7B) vocalizations significantly (n = 5, P < 0.05-0.01, compared to CRF alone at 15 min, Bonferroni posttests). KT5720 also inhibited the effects of CRF on withdrawal thresholds (Figure 7C, n = 5, P < 0.001, compared to predrug baseline, Bonferroni posttests). In contrast, co-administration of a PKC inhibitor (GF109203x, 100 μM) had no significant effect on CRF-enhanced vocalizations (Figure 7D, E, n = 6) and spinal reflexes (Figure 7F, n = 5). We showed previously that inhibitors of PKA and PKC alone have no effect on vocalizations and spinal reflexes in normal animals [45]. These control experiments were not repeated here.

### Behavioral effects of CRF are independent of HPA axis function

**Figure 8:**
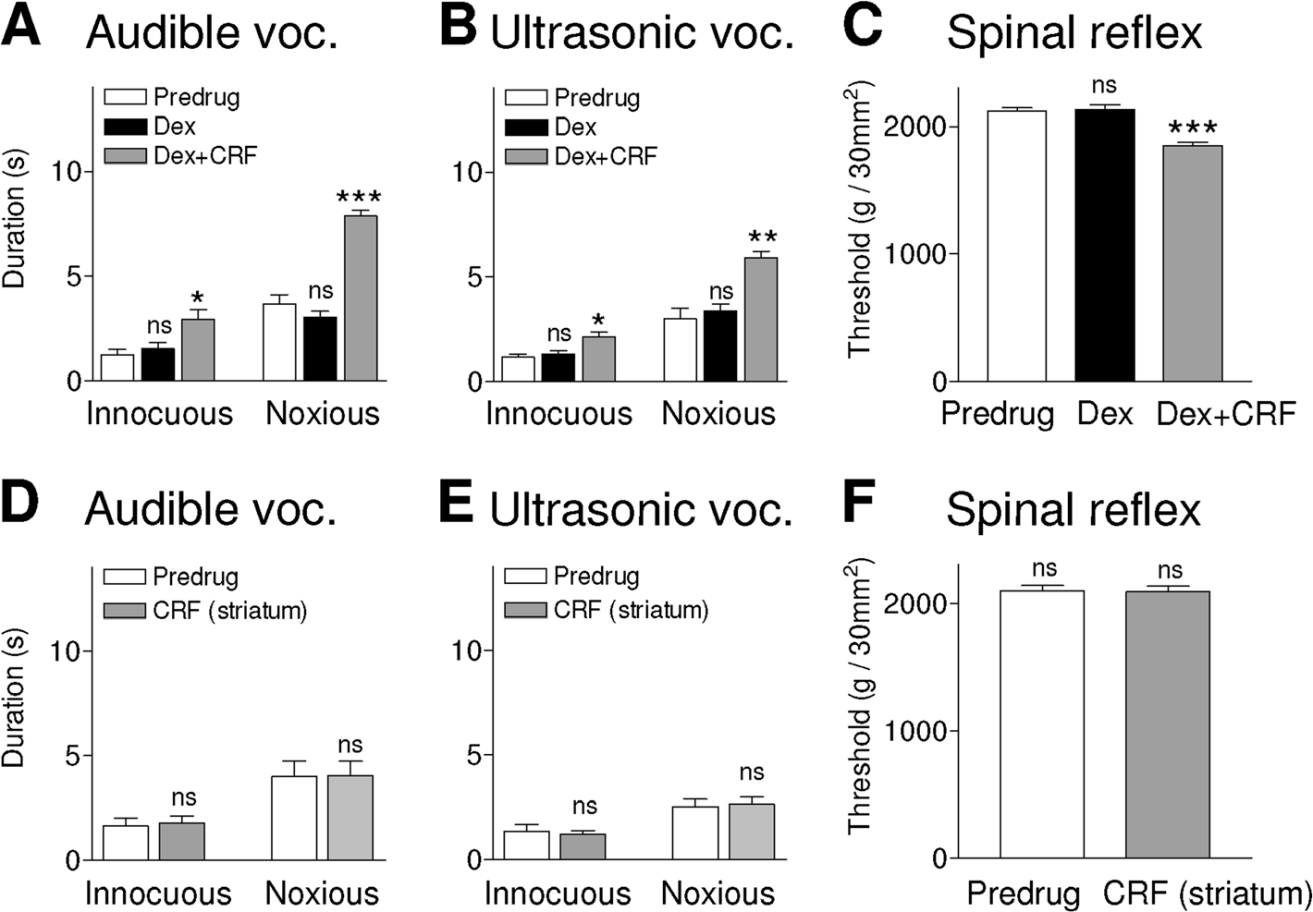
**HPA axis function and placement controls.** (**A-C**) To suppress HPA axis function rats were pretreated with dexamethasone (Dex, 0.05 mg/kg, subcutaneously) 2 hours prior to CRF administration into the CeLC [50,51]. Injection of Dex had no significant (ns) effect on audible and ultrasonic vocalizations and spinal reflex thresholds measured 15 min before CRF administration (n = 6 rats). Administration of CRF (1 μM, concentration in the microdialysis probe; 15 min) into the CeLC following pretreatment with Dex had significant facilitatory effects (n = 5 rats). (**D-F**) Administration of CRF (1 μM, concentration in the microdialysis probe; 15 min) into the adjacent striatum as a placement control had no significant effect on vocalizations and reflex thresholds (n = 6). Bar histograms show means ± SEM. *,**,*** P < 0.05-0.001, compared to Dex alone; ns (not significant) P > 0.05, compared to predrug; Bonferroni posttests.

CRF can act as a neuromodulator through extrahypothalamic mechanisms independently of HPA axis functions [1-4,8,11,13,49]. To determine if this is the case in amygdala-mediated pain modulation we used a well-established experimental approach, the so-called dexamethasone (Dex) suppression test (DST), in which pretreatment with a low dose of Dex (0.05 mg/kg, subcutaneously) suppresses the pituitary-adrenal response to CRF and inhibits plasma corticosterone increase [50,51]. Using this protocol we pretreated rats with dexamethasone (0.05 mg/kg, subcutaneously) 2 hours prior to CRF administration into the CeLC. Injection of Dex had no significant effect on audible and ultrasonic vocalizations and spinal reflex thresholds measured 15 min before CRF administration (n = 6 rats; Figure 8A-C). Pretreatment with Dex did not block the facilitatory effects of CRF (1 μM, concentration in the microdialysis probe; 15 min) that were statistically significant (P < 0.05-0.001, compared to Dex alone, Bonferroni posttests, n = 5 rats; Figure 8A-C). The data suggest that HPA axis function is not required for amygdala CRF to modulate pain-like behaviors.

### Placement controls and histology

**Figure 9:**
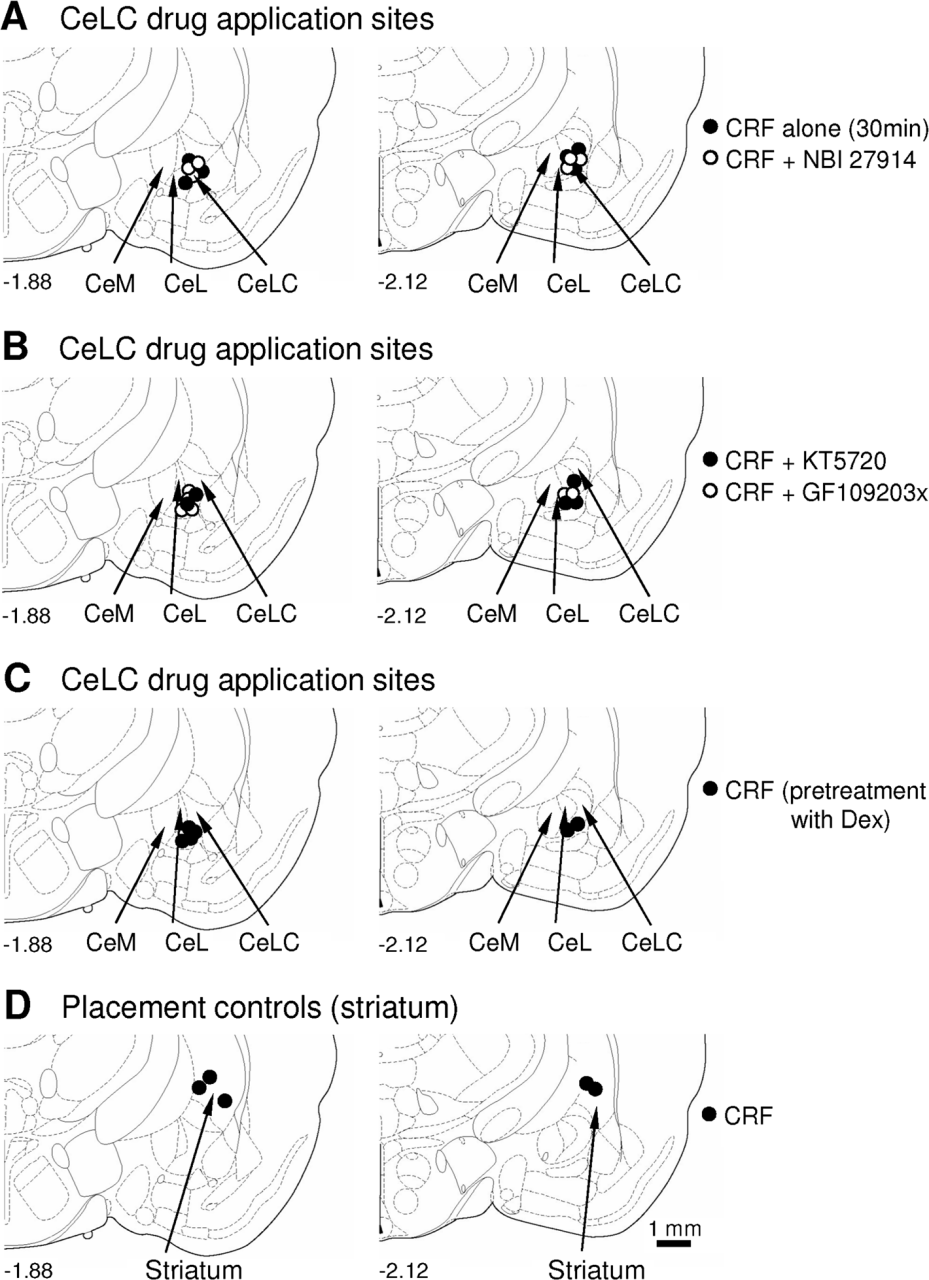
**Histological verification of drug application sites.** Diagrams adapted from Paxinos and Watson [75] show coronal sections through the right hemisphere at different levels posterior to bregma (−1.88 and −2.12). Next to each diagram is shown in detail the medial (CeM), lateral (CeL) and latero-capsular (CeLC) divisions of the central nucleus of the amygdala. Each symbol indicates the location of the tip of one microdialysis probe. The boundaries of the different amygdala nuclei are easily identified under the microscope (see Figure 1 in [29]). (**A**) Drug application sites for experimental data shown in Figure 6. (**B**) For data in Figure 7. (**C**) For data in Figure 8A-C. (**D**) For data in Figure 8D-F.

As a control for drug diffusion, CRF (1 μM, concentration in the microdialysis probe; 30 min) was administered into the adjacent striatum as in our previous studies [43,45,46,48]. CRF in the striatum had no significant effect on vocalizations and spinal reflexes (n = 6 rats; Figure 8D-F). Drug application sites into the CeLC and striatum were verified histologically (see Methods). Figure 9 shows the position of the tips of the microdialysis probes for the different sets of behavioral experiments (see Figures 6, 7, 8).

## Discussion

The novelty of this study is the correlation of electrophysiological effects of CRF in the amygdala with pain-like behaviors under normal conditions in the absence of injury or disease. We show that CRF administered to the CeLC, a brain area that plays a critical role in the emotional-affective component of pain [21,22], enhances or triggers nocifensive and affective behaviors by increasing synaptic transmission and neuronal output through postsynaptic CRF1 receptor-mediated PKA activation.

CRF facilitated synaptic transmission at the PB-CeLC synapse that provides unfiltered nociceptive information from spinal cord and brainstem to the CeLC and undergoes plasticity in different pain models [23-30]. Increased transmission at the PB-CeLC synapse correlates with pain- and anxiety-like behaviors [21,22]. Analysis of miniature EPSCs indicated a post- rather than presynaptic action of CRF. CRF also increased spike firing, suggesting increased neuronal output. We did not attempt to determine the ionic basis for this cellular effect. Our previous studies using CRF receptor antagonists in the arthritis pain model implicated Kv3-type potassium channels in the effects of endogenous activation of CRF1 receptors; Kv3 channel regulate firing rate through action potential repolarization [29]. However, a number of other effects of CRF on electrophysiological properties of amygdala neurons have been described, including inhibition of the slow afterhyperpolarizing potential (AHP) following evoked repetitive firing, mixed effects on the medium AHP [39], and increase of R-type voltage-gated calcium channels [52]. Importantly, non-accommodating Type A neurons recorded in this study only show a medium AHP whereas the slow AHP is characteristic of accommodating Type B neurons [40]. Therefore, the mechanisms of any membrane effects of CRF are likely complex and warrant the detailed analysis in a separate study.

The effects of CRF could be blocked with a CRF1 but not CRF2 receptor antagonist, demonstrating the presence of functional CRF1 receptors in the CeLC under normal conditions. This is not trivial because CRF1 antagonists had no effect on their own under normal conditions in previous studies from our group [29,37] and others [36]. The lack of involvement of CRF2 receptors in CRF-induced synaptic facilitation may be due to the lower affinity of CRF for this receptor type [3,53] or different expression levels of CRF1 and CRF2 receptors in the synaptic circuit studied here [54,55]. CRF2 receptor mRNA expression is more restricted than that of CRF1, at least under normal conditions; highest levels of CRF2 receptor mRNA in the brain are found within the lateral septum, ventromedial hypothalamus and choroid plexus, while medial and posterior cortical nuclei of the amygdala show moderate expression levels [55,56].

CRF effects were largely blocked by inhibition of PKA but not PKC. CRF receptors can couple to a number of G-proteins to activate a variety of intracellular signaling pathways, and PKA and PKC appear to play particular important roles [6]. PKA is a critical contributor to pain-related plasticity of CeLC neurons in the arthritis model [28,45]. A consequence of PKA activation is the NR1 subunit phosphorylation of NMDA receptors in the CeLC and increased NMDA receptor-mediated synaptic transmission in the arthritis pain model [27,28]. The present study found that CRF can increase a latent NMDA component through CRF1 receptor-mediated PKA activation, suggesting that CRF can engage processes similar to those that generate synaptic plasticity in pain models such as arthritis.

Interestingly, synaptic plasticity in the CeLC in neuropathic pain does not depend on NMDA receptors [24] although NMDA receptor antagonists are effective in the CeA in reducing nocifensive and affective pain behaviors in a neuropathic pain model [57]. CRF and CRF mRNA are increased in CeA neurons in neuropathic pain [33] but increasing endogenous CRF in the CeA with a CRF-binding protein inhibitor had mixed effects in neuropathic pain, facilitating nocifensive responses while attenuating emotional-affective behaviors [36]. The data may suggest that NMDA and CRF receptors engage different elements of the intra-amygdala circuitry in neuropathic pain, acting for example on inhibitory systems that modulate CeA processing [30] and neuropathic pain responses [58] and can be engaged by CRF1 [59] or CRF2 [29] receptors. Here we focused on the modulation of excitatory transmission at the PB-CeLC that correlates positively with pain behaviors [21,22] although this study does not rule out additional sites of action of CRF in the amygdala network that should be explored.

In our study, the electrophysiological effects of CRF correlated with behavioral consequences. CRF increased audible and ultrasonic vocalizations, which represent supraspinally organized nocifensive and affective responses to aversive stimuli [47], and decreased thresholds for spinal reflexes. The results are consistent with the concept that increased CeLC output, here induced by CRF, facilitates spinal and supraspinal behaviors. It remains to be determined if this is accomplished through descending facilitation or disinhibition [21]. Non-accommodating Type A neurons in the CeA project to brainstem and forebrain areas involved in the expression of aversive behaviors and pain modulation, including the periaqueductal gray. These brainstem projections arise not only from medial but also lateral regions of the CeA and involve strong interconnections between CeLC and substantia innominata [21,40,60,61]. Lateral CeA projection neurons contain a number of neuropeptides, including CRF, neurotensin and somatostatin, and the latero-capsular region is the major site of extrahypothalamic CRF expression [12,33]. Direct brainstem projections from CeA can be glutamatergic [62] but CRF-containing CeA neurons also include a population of GABergic neurons [63,64]. Therefore, CeLC output can activate descending facilitation or inhibit descending inhibition (dis-inhibition) to produce the behavioral effects of CRF observed in our study. The results of our experiments in which the HPA axis response was suppressed with dexamethasone pretreatment distinguish this neuromodulatory function of CRF from its role as a stress hormone.

The significance of our findings is that increasing CRF in the amygdala can trigger pain-like behaviors in normal animals and these behavioral effects correlate with increased neuronal activity in the CeLC. Pain arising from altered brain functions in the absence of tissue injury represents a clinically important concept that could explain pain or increased pain sensitivity in conditions of anxiety, depression, or addiction such as alcohol dependence [4,9,13,65] that involve the CRF system in the amygdala. With regard to pain processing and pain modulation, exogenous application of CRF (this study) and endogenous release of CRF measured indirectly with a CRF1 receptor antagonist in our previous studies [29,37] appear to have similar facilitatory effects.

Some technical aspects of our study deserve consideration. Choice of drugs at appropriate concentrations is critical for pharmacological validity. We used selective compounds at concentrations that are well established in the literature [7,66-68] and in our own previous studies [29,35,37,38,45]. The fact that a selective CRF1 antagonist inhibited the synaptic and behavioral effects of CRF clearly implicates CRF1 receptors. The lack of effect of a CRF2 receptor antagonist was not due to an insufficient drug concentration because the same concentration produced facilitatory effects in a pain model in our previous study [29]. Likewise, the PKC inhibitor was used at a concentration near the high end of the selective range to ensure the lack of effect on CRF functions was not due to an insufficient concentration. A lower concentration of this inhibitor has been shown to be effective in modulating G-protein coupled receptor function [69]. Additional caveats need to be considered for drug application by microdialysis. While the concentration of the drug in the microdialysis fiber is known, the drug dose administered can only be estimated. Comparative data from our previous microdialysis and in vitro studies using CRF receptor compounds [29,35,37,70] indicate that the tissue concentration is at least 100 times lower than in the microdialysis probe due to the concentration gradient across the dialysis membrane and diffusion in the tissue. Therefore, drugs were dissolved in ACSF at a concentration 100 times that predicted to be needed. Microdialysis was chosen for drug delivery because it offers several advantages, including continued drug delivery and steady state levels without a volume effect [71]. Differential effects of CRF1 and CRF2 receptor antagonists and PKA and PKC inhibitors here and in our previous studies [29,35,37,38,45] argue against non-selective drug effects at the concentrations used. Placement control experiments suggest that the drugs did not spread beyond a distance of 1 mm around the tip of the microdialysis probe, which is consistent with our previous estimates [35,37,38,70]. The distance between the tips of the microdialysis probes in the CeLC (effective drug administration site) and striatum (ineffective control site) is about 2 mm. The striatum was selected as in our previous studies [43,45,46,48] because it is located adjacent (dorsolateral) to the CeLC but does not project directly to the CeLC [72]. Desensitization of CRF effects needs to be considered. However, the persistence of CRF effects in the presence of a CRF2 antagonist (Figure 1E) and PKC inhibitor (Figure 4E) and during prolonged drug application (Figure 4C) argues against the loss of effectiveness, e.g., due to desensitization, as an explanation for the inhibitory effect of the CRF1 antagonist and PKA inhibitor.

## Conclusion

Non-pain-related activation of CRF1 receptors in the amygdala can trigger pain-responses in normal animals through a mechanism that involves PKA-dependent synaptic facilitation in CeLC neurons independent of HPA axis function. The study contributes novel insight into CRF functions in the brain and into brain mechanisms of pain. The results suggest that conditions of increased amygdala CRF levels can trigger pain in the absence of any tissue pathology or disease state.

## Methods

Male Sprague Dawley rats (150–350 g) were housed in a temperature controlled room and maintained on a 12 h day/night cycle. Water and food were available *ad libitum*. On the day of the experiment, rats were transferred from the animal facility and allowed to acclimate to the laboratory for at least 1 h. All experimental procedures conform to the guidelines of the International Association for the Study of Pain (IASP) and of the National Institutes of Health (NIH) and were approved by the Institutional Animal Care and Use Committee (IACUC) at the University of Texas Medical Branch (UTMB).

### Electrophysiology

#### Brain slice preparation

Coronal slices (300–500 μm) containing the CeLC were obtained from normal untreated rats (150–230 g) as previously described [30,42,43] for recent references see [46]. Rats were decapitated without the use of anesthesia to avoid chemical contamination of the tissue. A single brain slice was transferred to the recording chamber and submerged in artificial cerebrospinal fluid (ACSF; 31 ± 1°C), which superfused the slice at ~2 ml/min. ACSF contained (in mM) NaCl 117, KCl 4.7, NaH_2_PO_4_ 1.2, CaCl_2_ 2.5, MgCl_2_ 1.2, NaHCO_3_ 25, and glucose 11. The ACSF was oxygenated and equilibrated to pH 7.4 with a mixture of 95% O_2_/5% CO_2_. Only one or two brain slices per animal were used, one neuron was recorded in each slice, and a fresh slice was used for each new experimental protocol. Numbers in the manuscript refer to the number of neurons tested for each parameter.

#### Patch-clamp recording

Whole-cell current- and voltage-clamp recordings were made from CeLC neurons using the “blind” patch technique or DIC-IR video-microscopy as described before for recent references see [30,42,43]. The boundaries of the different amygdalar nuclei are easily discerned under light microscopy see Figure 1 in [29]. Recording pipettes (3–5 MΩ tip resistance) were made from borosilicate glass (1.5 mm and 1.12 mm, outer and inner diameter, respectively; Drummond, Broomall, PA) using a Flaming-Brown micropipette puller (P-80/PC, Sutter Instrument Co., Novato, CA). Electrodes were filled with intracellular solution containing (in mM): 122 K-gluconate, 5 NaCl, 0.3 CaCl_2_, 2 MgCl_2_, 1 EGTA, 10 HEPES, 5 Na_2_-ATP, and 0.4 Na_3_-GTP; pH is adjusted to 7.2-7.3 with KOH and osmolarity to 280 mOsm/kg with sucrose. Data acquisition and analysis was done using a dual 4-pole Bessel filter (Warner Instr.), low-noise Digidata 1322 interface (Molecular Devices), Axoclamp-2B amplifier (Molecular Devices), Pentium PC, and pClamp10 software (Molecular Devices). Signals were low-pass filtered at 1 kHz and digitized at 5 kHz. Headstage voltage was monitored continuously on an oscilloscope to ensure precise performance of the amplifier. High GΩ seal and low series (<20 MΩ) resistances were checked throughout the experiment (using pClamp10 membrane test function) to ensure high-quality recordings. If series resistance (monitored with pClamp190 software) changed more than 10%, the neuron was discarded. Neurons were recorded at −60 mV except when NMDA receptor-mediated responses were studied.

#### Synaptic transmission

**Monosynaptic EPSCs** were evoked in CeLC neurons by focal electrical stimulation (Grass S88 stimulator) of inputs from the PB. For stimulation of the PB-CeLC synapse, a concentric bipolar stimulating electrode (SNE-100, Kopf Instr.; 22 kW). was positioned under microscopic control on the fiber tract that runs dorsomedial to the CeA and ventral to but outside of the caudate-putamen [25,29]. In the vicinity of this tract, no afferents to the CeA other than from lateral PB have been described [19,20]. Electrical stimuli (150 μs square-wave pulses) were delivered at low frequencies (< 0.25 Hz). Input–output functions were obtained by increasing the stimulus intensity in 100 μA steps. For evaluation of a drug effect on synaptically evoked responses, the stimulus intensity was adjusted to 75-80% of the intensity required for orthodromic spike generation. EPSCs were recorded at −60 mV in the presence of bicuculline (30 μM) except for the study of NMDA receptor-mediated EPSCs, which were recorded at +20 mV in the presence of bicuculline (30 μM) and NBQX (20 μM) as in our previous studies [46].

**Miniature EPSCs** (mEPSCs, in TTX 1μM) were measured as described previously [42,46]. A fixed length of traces (5 min) was analyzed for frequency and amplitude distributions using MiniAnalysis program 5.3 (Synaptosoft, Decatur, GA). The root mean square (RMS) of the background noise was computed for each set of data. Detection threshold for an event was set to 3–4 times the RMS value. Peaks were detected automatically, but each detected event was then visually inspected to prevent the inclusion of false data.

#### Drug application

Drugs (see below, “Drugs”) were applied by gravity-driven superfusion of the brain slice in the ACSF (~2 ml/min). Solution flow into the recording chamber (1 ml volume) was controlled with a three-way stopcock.

### Behavioral tests

Adult male Sprague–Dawley rats (250–350 g) were used in all experiments.

#### Spinal reflexes

Thresholds of hindlimb withdrawal reflexes evoked by mechanical stimulation of the knee joint were measured as described previously [47]. Mechanical stimuli of continuously increasing intensity were applied to the knee joint by means of a forceps equipped with a force transducer, whose calibrated output was amplified and displayed in grams on a liquid crystal display screen. Withdrawal threshold was defined as the minimum stimulus intensity that evoked a withdrawal reflex.

#### Vocalizations

Audible and ultrasonic vocalizations were recorded and analyzed as described previously [43,70,73]. The experimental setup (U.S. Patent 7,213,538) included a custom-designed recording chamber, a condenser microphone (audible range, 20 Hz to 16 kHz) connected to a preamplifier, an ultrasound detector (set to record ultrasonic vocalizations in the range of 25 ± 4 kHz), filter and amplifier (UltraVox 4-channel system; Noldus Information Technology). Vocalizations in the audible and ultrasonic ranges were recorded simultaneously but with different microphones (condenser microphone and bat detector, respectively) connected to separate channels of the amplifier. Data acquisition software (UltraVox 2.0; Noldus Information Technology) monitored the occurrence of vocalizations within user-defined frequencies and recorded the number and duration of digitized events (audible and ultrasonic vocalizations). The computerized recording system was set to suppress non-relevant audible sounds (background noise) and to ignore ultrasounds outside the defined frequency range. Animals were placed in the recording chamber for acclimation 1 h before the vocalization measurements.

Brief (15 s) innocuous (500 g/30 mm^2^) and noxious (2000 g/30 mm^2^) mechanical stimuli were applied to the knee, using a calibrated forceps. Stimulus intensities of 100–500 g/30 mm^2^ applied to the knee and other deep tissue are considered innocuous because they do not evoke hindlimb withdrawal reflexes in awake rats and are not felt to be painful when tested on the experimenters. Pressure stimuli >1500 g/30 mm^2^ are noxious because they evoke hindlimb withdrawal reflexes in awake rats and are distinctly painful when applied to the experimenters [47]. The total duration of vocalizations (arithmetic sum of the duration of individual events) was recorded for 1 min, starting with the onset of the mechanical stimulus.

#### Drug application by microdialysis in awake animals

As described in detail previously [43,70], a guide cannula was implanted stereotaxically the day before behavioral measurements, using a stereotaxic apparatus (David Kopf Instr.). The animal was anesthetized with pentobarbital sodium (Nembutal, 50 mg/kg, i.p.) and a small unilateral craniotomy was performed at the sutura frontoparietalis level. The guide cannula was implanted on the dorsal margin of the CeLC in the right hemisphere, where pain-related changes have been shown [74], using the following coordinates (in mm): CeLC, 2.0 caudal to bregma, 4.0 lateral to midline, depth 7.0. In some experiments a guide cannula was implanted into the striatum as a placement control, using the following stereotaxic coordinates: 2.0 mm caudal to bregma; 4.0 mm lateral to midline; depth of tip 5.0 mm. The cannula was fixed to the skull with dental acrylic (Plastics One). Antibiotic ointment was applied to the exposed tissue to prevent infection.

On the day of the experiment, a microdialysis probe (CMA12; CMA/Microdialysis Inc.; 20 kD cut-off, membrane length 2 mm) was inserted through the guide cannula so that the probe protruded by 2 mm. The probe was connected to an infusion pump (Harvard Apparatus) and perfused with ACSF (oxygenated and equilibrated to pH = 7.4). Drugs (see below, “Drugs”) were dissolved in ACSF on the day of the experiment at a concentration 100-fold that predicted to be needed based on data from our previous microdialysis and in vitro studies because of the concentration gradient across the dialysis membrane and diffusion in the tissue [29,35,37,70]. Numbers in the manuscript refer to drug concentrations in the microdialysis fiber. Drugs were applied by microdialysis at a rate of 5 μl/min for at least 20 min to establish equilibrium in the tissue. Before each drug application, ACSF was pumped through the fiber for at least 1 h to establish equilibrium in the tissue.

#### Histological verification of drug administration sites

The position of the microdialysis probe in the CeA or striatum (placement control) was confirmed histologically. At the end of each behavioral experiment, the animal was euthanized by decapitation using a guillotine (Harvard Apparatus Decapitator). The brain was removed and submerged in 10% formalin. Tissues were stored in 20% sucrose before they were frozen sectioned at 50 μm. Sections were mounted on gel-coated slides, stained with hematoxylin and eosin (H&E) and cover-slipped. Positions of the microdialysis fibers were identified under the microscope and plotted on standard diagrams adapted from Paxinos and Watson [75].

### Drugs

The following drugs were used: Corticotropin releasing factor (human, rat; CRF) was purchased from Bachem. 5-chloro-4-(N-(cyclopropyl)methyl-N-propylamino)-2-methyl-6-(2,4,6-trichlorophenyl) amino-pyridine (NBI 27914; CRF1 receptor antagonist) [66] was purchased from Tocris Bioscience. Cyclo(31–34) [D-Phe11,His12,CαMeLeu13,39, Nle17, Glu31, Lys34] Ac-Sauvagine(8–40) (astressin-2B; CRF2 receptor antagonist) [67,68] was purchased from Sigma-Aldrich. (9*R*,10*S*,12*S*)-2,3,9,10,11,12-hexahydro-10-hydroxy-9-methyl-1-oxo-9,12-epoxy-1*H*-diindolo[1,2,3-*fg*:3’,2’,1’-*kl*pyrrolo[3,4-*i*[1,6]benzodiazocine-10-carboxylic acid, hexyl ester (KT5720; membrane-permeable potent and selective PKA inhibitor) [76]; 2-[1-(3-dimethylaminopropyl)indol-3-yl]-3-(indol-3-yl) maleimide (GF109203x; membrane-permeable potent and selective PKC inhibitor) [77]; 2,3-dioxo-6-nitro-1,2,3,4-tetrahydrobenzo[f]quinoxaline-7-sulfonamide disodium salt (NBQX; non-NMDA receptor antagonist); DL-2-amino-5-phosphonopentanoic acid (AP5; NMDA receptor antagonist); *R*-(R*,S*)]-6-(5,6,7,8-tetrahydro-6-methyl-1,3-dioxolo[4,5-g]isoquinolin-5-yl)furo[3,4-e]-1,3-benzodioxol-8(6H)-one (bicuculline; GABA_A_ receptor antagonist); and dexamethasone (to suppress HPA axis function [51]) were purchased from Tocris Bioscience. Selectivity and target concentrations have been established in our previous studies [29,37,38,45]. Drugs were dissolved in ACSF on the day of the experiment. ACSF served as vehicle control in all experiments.

### Statistical analysis

All averaged values are given as the mean ± SEM. Statistical significance was accepted at the level P < 0.05. GraphPad Prism 3.0 software (Graph-Pad Software, San Diego, CA) was used for all statistical analysis. For multiple comparisons, one-way or two-way ANOVA was used with appropriate posttests as indicated. Paired student t-test was used to compare two sets of data that follow Gaussian distribution and have similar variances. Concentration–response curves were obtained by non-linear regression analysis using the formula *y* = *A* + (*B* − *A*)/[1 + (10^*C*^/10^*X*^)^*D*^], where *A* is the bottom plateau, *B* top plateau, *C* = log(IC_50_), and *D* is the slope coefficient (GraphPad Prism software). Kolmogorov-Smirnov test was used for cumulative distribution analysis of mEPSCs (MiniAnalysis program 5.3, Synaptosoft Inc.).

## Abbreviations

BLA: Basolateral nucleus of the amygdala; CeA: Central nucleus of the amygdala; CeLC: Latero-capsular division of the CeA; CRF: Corticotropin-releasing factor; Dex: Dexamethasone; EPSC: Excitatory postsynaptic current; LA: Lateral nucleus of the amygdala; mEPSC: Miniature EPSC; PB: Parabrachial area.

## Competing interests

The authors declare that they have no competing interests.

## Authors’ contributions

GJ carried out electrophysiological and behavioral experiments, analyzed data and prepared figures. YF performed electrophysiological experiments, analyzed data and prepared figures. HA performed behavioral experiments, analyzed data and prepared figures. VN conceptualized the hypothesis, coordinated the study, designed and supervised the experiments, directed the data analysis, and finalized the manuscript. All authors read and approved the manuscript.
